# Communities of Care Approach: Developing a Place-based Model of Care and Building Partnerships in the Communities in Central Singapore

**DOI:** 10.5334/ijic.7727

**Published:** 2024-04-09

**Authors:** Wei TING CHEN, Sing YONG LIM, Shermaine How, Woan SHIN TAN, Ian Yi Onn Leong

**Affiliations:** 1 Division for Central Health, Tan Tock Seng Hospital, Singapore; 2 Health Services & Outcomes Research, National Healthcare Group, Singapore

**Keywords:** local communities, care delivery, partnership, neighbourhood

## Abstract

The population in Singapore is ageing, adding pressure to community care as the health and social needs of its residents increase. This has accelerated the pace at which Regional Health Systems adopt and deliver its population health strategies from early prevention, chronic disease management, crisis care to end-of-life care. To this end, the Central Health Integrated Care Network (ICN) began its journey to develop Communities of Care (CoCs) with other health and social care partners to meet the needs of residents in the Central Zone of Singapore. This paper describes the processes and steps taken by Central Health ICN to build partnerships with other agencies and organisations to build place-based models of care in the local neighbourhoods. The faciliating factors and the barriers faced in the implementation of CoCs were described to allow sharing of such learnings on large scale change. Strategies in overcoming some of the challenges were also presented to demonstrate the iterative processes required in building integrated place-based models of care to meet the needs of the residents in different communities.

## Introduction

Singapore has an ageing population. The proportion of residents aged 65 and above has increased from 10.4% in 2011 to 18% in 2021 [1]. The population will become ‘super-aged’ by 2026, with 24% projected to be aged 65 and above by 2030. Chronic disease prevalence has similarly been increasing over time. The proportion of residents with hypertension has grown from 24% in 2017 to 36% in 2020. The prevalence of high cholesterol and obesity has also grown from 36% to 39% and from 9% to 11% respectively over the same period [2]. Social determinants of health are increasingly recognised as important drivers of health and well-being, warranting the shift towards better integration of both health and social care to support ageing in place [3,4]. This is especially so in Singapore, where health care delivery has traditionally been episodic and facility-based. Each service provider comes into the picture only when the need arises. This would not adequately meet the diverse needs of a population that is ageing and has a heavy chronic disease burden [3]. From the patient’s point of view, there would be multiple providers and touch points, rendering it challenging for them to navigate the care system and making care sub-optimal.

Much have been already written on the principles of integrating health and social services. Most notably, Leutz (1999) wrote about how to integrate medical and social care. One of his key recommendations focused on the importance of developing systems for integrating, coordinating and linking services for persons with needs [5]. The World Health Organisation (WHO) framework on integrated people-centred health services (IPCHS) further called for a shift towards focusing on empowering people and communities, and to collaborate across organisations and providers [6]. Singapore has similarly pivoted towards population health. Three Regional Health Systems (RHS) have been established as population health managers in the Western, Central-North and South-Eastern regions of Singapore, representing a shift from managing patients only in times of acute sickness to managing the health of a population [7]. The launch of HealthierSG as a national initiative further complements this by encouraging Singaporeans to have a health plan anchored with a primary care doctor and supported by community partners to manage social prescription for better integration of care across the life course [7].

**Figure 1:**
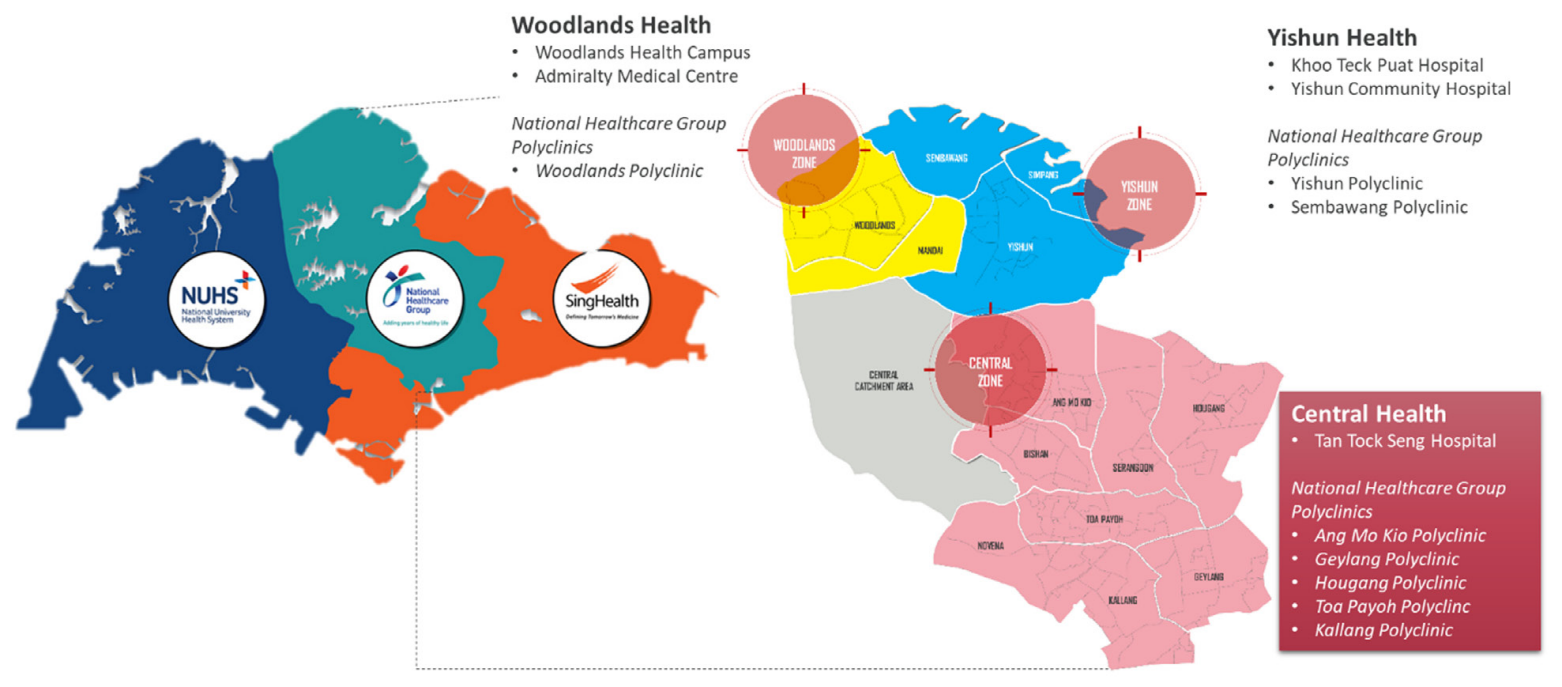
Overview of Singapore’s Public Healthcare Clusters and National Healthcare Group’s Integrated Care Networks (ICN) in the Central-North region.

While the RHSs embrace the principles of care integration, they mainly operate at the strategic-level to address concerns in health and social care. Smaller place-based partnerships within the RHSs are required to bring multi-provider teams together to deliver personalised care that is localised to the specific needs of the community at the neighbourhood level. The National Healthcare Group, as the Central-North RHS, aims to scale this through building place-based Communities of Care (CoC), localised networks of health and social partners to deliver and increase accessibility to integrated care services within their communities. Specifically within the Central Zone, these are driven through the efforts of the Central Health Integrated Care Network (ICN) (Figure 1), an alliance of providers (or integrated care provider) that is involved in the planning and development of cross-sector programmes to address the spectrum of care needs of the local population [3,8].

This paper aims to describe the conceptualisation and development of CoC as a place-based health and social care integration model in the Central Zone.

## Problem Statement

In 2022, 922,760 residents or 23% of Singapore’s population reside within the Central Zone. The challenge of addressing a growing national chronic disease burden, and a surge in frailty is even more pronounced in this region where approximately 21% of these residents were aged 65 in 2022 compared to the national average of 17% [9] and were of lower socio-economic status. The median household income in the Central Zone ranged between USD$4,500–5,300 compared against the national median of USD$5,300–6,000 [10].

System-level changes and initiatives have been implemented over the years to better integrate health and social care in Singapore in order to more holistically address the needs of the population. Since 2013, planning and policy oversight of health and social support services were consolidated in phases under the Ministry of Health (MOH) [11]. In spite of this, programmes and projects continued to operate as in-silo [12]. Primary care and community healthcare providers, as independent operators, have limited incentives to integrate with other non-governmental organisations (NGOs) [12]. Resources and capabilities of community providers also differ. Larger NGOs and private operators tended to be able to provide more integrated care services as compared to smaller organisations, having better corporate support and funding [12]. As such, care remains fragmented across various neighbourhoods. Adding to these challenges, there is also a population of vulnerable older persons with low social support and who are at risk of social isolation living in rental flats and studio apartments [11]. A previous survey of this population showed that more than half of them rarely access activities at drop-in centres near them and of this sub-group, approximately 10% had three or more difficulties in Activities of Daily Living (ADLs) [11]. Therein lies the impetus to quicken the operationalisation of integrated care models in the neighbourhoods throughout Central Zone to match the needs of the rapidly ageing population and to extend beyond illness care to include preventive and wellness care.

There have been several notable projects implemented in the community for care integration. The Community for Successful Ageing (ComSA) project that was launched in 2015 is one such example that took a ground-up approach to introduce the Patient-Centred Medical Home (PCMH) model in a neighbourhood (Whampoa) in the Central Region of Singapore. This project established an ecosystem consisting of multidisciplinary health and social services, civic empowerment and participation, and policy making [13]. It further presented considerations for how biopsychosocial components of community programmes could interface and build on one another to serve the different aspects of care [14].

Considering the influence of local community planning and design on health and well-being, another key research on place-based care was undertaken to understand the development of healthy communities using an evidence-based approach [15]. A mixed-method research approach was undertaken to study the activities and interactions in the communities and the design of the space and communities in another neighbourhood (Chong Pang), culminating in the set-up of Wellness Kampung in the Northern region of Singapore in 2015. This research provided important insights into how formal and informal health and social care integration could promote health and well-being in the community.

While these past projects had provided us with insights into care integration, they have remained largely contextualised at the local level and within single communities. The systematic implementation of integrated care models at a larger scale and across communities with their own unique health and social needs has not yet been studied in detail in Singapore. As we took in the learnings from these projects and evolved our population health approach to integrate care across health and social care sectors, we established a framework, processes and enablers that would be required to develop large scale changes across varying neighbourhoods. Here we share our iterative process and learning journey in building CoCs. Insights gained through the large scale implementation of 47 CoCs over the last two years will be shared and critically examined for development of similar models, especially in the Singapore context.

## Description of a CoC

### Evolution of Place-Based Care

“Place-based partnerships are collaborative arrangements between organisations responsible for arranging and delivering health and care services and others with a role in improving health and well-being” [16]. They are intended to bring together the combined resources of a local region more effectively. This is important as residents can have differing needs, and those with chronic conditions frequently require a range of services offered by a network of different providers collaborating across organisations. With an ageing population, it has also become all the more important to adopt a life-course approach supported by this network to enable ageing in place. Within the Central Zone, this is operationalised by establishing CoCs to support the health and social care needs of residents within a geographically defined locality. Activities and collaborations within the CoCs are broadly categorised based on the National Healthcare Group (NHG)’s life course approach framework for population health management, comprising five segments of care – Living Well, Living with Illness, Crisis and Complex Care, Living with Frailty, and Leaving Well [17].

Central Health ICN’s initial place-based care efforts started in 2016 with the establishment of three Community Health Posts in one neighbourhood (Toa Payoh) as part of a funded programme by MOH and with a focus on seniors. This programme saw the set-up and co-location of Tan Tock Seng Hospital’s (TTSH) Community Health Teams (CHT)^1^ within social communal spaces, such as Senior Activity Centres (SACs) and local citizens’ activity centres, to provide lifestyle advice to residents with pre-onset or early stage diabetes for better health management. This was the foundation upon which subsequent collaborations were built, expanding the accessiblity and range of health and social care services and programmes as more community-based partners came onboard as part of the local neighbourhood network.

The nature of our partherships have evolved from co-location of services to established bi-lateral referral workflows for increased access to care. In 2019, we embarked upon a housing block mapping exercise which sought to study the demographics and the needs of residents through sharing of data among providers. This allowed the network to better profile the neighbourhood, identify and perform targeted outreach to vulnerable residents, and develop shared care plans and workflows for co-management, representing a shift towards joint efforts in caring for the neighbourhood [18].

Lessons on the key processes and systems enabling community care were also gleaned from other more developed models. The Buurtzorg Nederland model provided insights on possible frameworks on how independent community nursing teams could work with other formal and informal carers to support care in the neighbourhood more effectively [19]. We adopted the use of the Omaha System standardised taxonomy to better structure and document person-centric care plans and enable more systematic data analysis of care outcomes [20]. We also took away key observations from the National Health System in the United Kingdom, including the importance of citizen engagement, how neighbourhood networks may be formed and organised, the involvement of the third sector, and working models with primary care [21]. The development of our CoCs were further informed by the following key principles conveyed by the Torbay and South Devon locality team: (i) person-centred care and helping residents to make positive sustainable changes to their lives; (ii) regular common platforms (i.e. ‘one big room’) to facilitate communication and exchange of perspectives amongst a multi-disciplinary team; and (iii) establish a high degree of trust within the locality team [22].

Through building upon these initial projects and coupled with learning from other models before ours, the Central Health ICN has established its framework for building CoCs.

**Figure 2:**
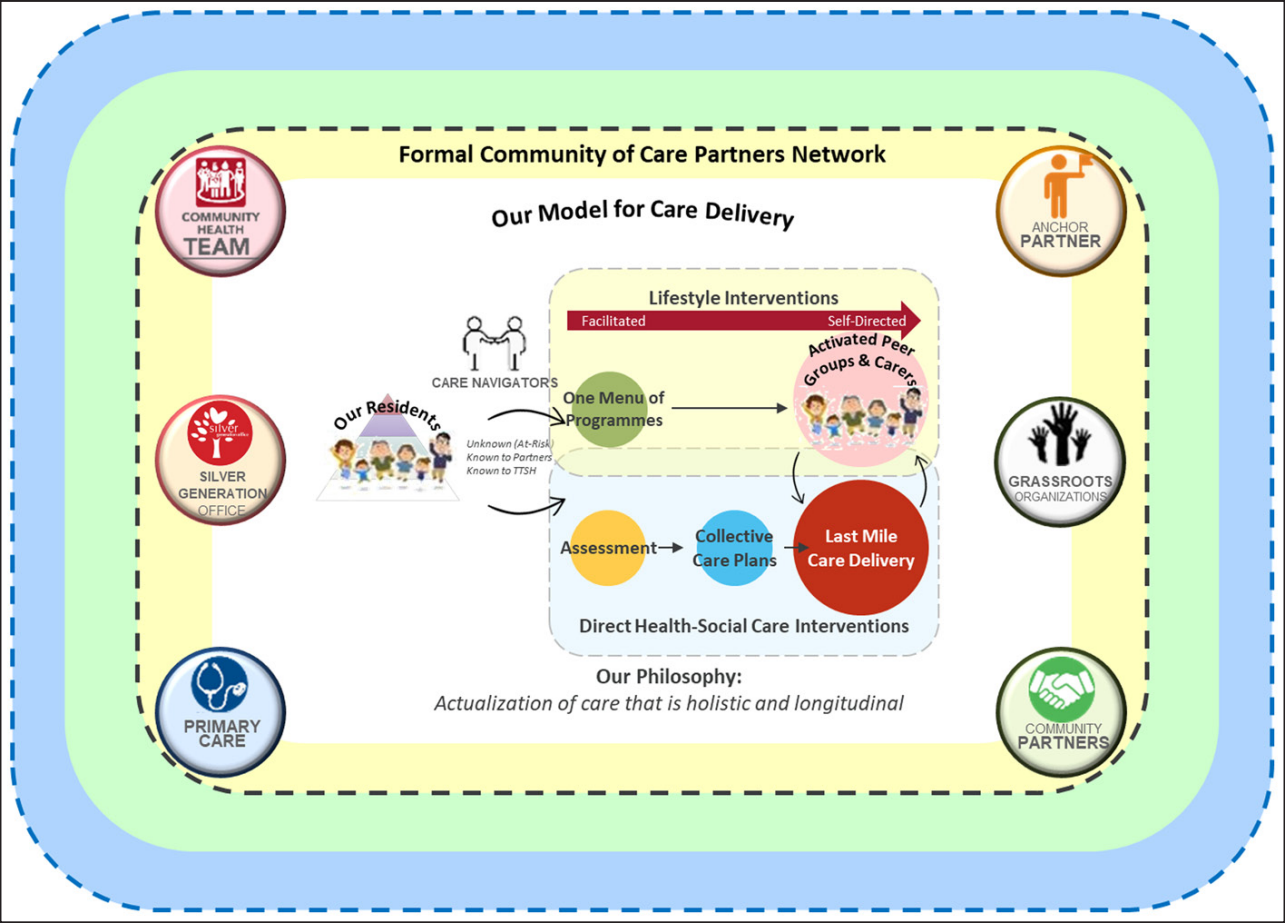
Central Health Community of Care Framework.

CoCs bring together a network of health and social partners to integrate and actualise community care in a neighbourhood, guided by Three ‘A’s: Ageing Well in Place; Activating for Health; and Anchoring Care with Partners (Figure 2). Each CoC comprises of the following components:

1. **A network of care partners**. Within each CoC, there is a main NGO (an anchor partner), often an operator of an Active Ageing Centre (AAC)^2^, that serves as a key community node for residents living in the neighbourhood [23]. It provides opportunities for residents to gather, engage with one another through a range of social activities and a touchpoint where residents may be able to seek help in linking up with a range of care and support services offered by other community partners. Health-related needs are managed by primary care partners including General Practice Clinics, Family Medicine Clinics and Polyclinics that provide each resident with one primary physician. TTSH CHT complements the anchor partner and primary care to form the fundamental bridge for health and social integration through co-developing and providing lifestyle and preventive care programmes that can be accessible in community spaces. For more vulnerable residents or those with more complex needs, CHT facilitates the co-development and co-management of health-social care plans. Finally, grassroots, social agencies such as the Silver Generation Office^3^, and other health agencies further extend the capabilities of the network by expanding the range of health and social services and programmes that can be provided through established cross-referral processes and collaborations.
2. **Common needs assessments**. The network of care partners engage in collaborative arrangements to reach out to residents residing within the local community to better understand their needs in order to provide more upstream interventions or recommendations on activities to ensure continued social engagement and health maintenance. Common assessment tools based on InterRAI are used to align understanding of the needs identified and follow-up pathways across the various partners.
3. **One Menu of Programmes**. The range of activities and programmes that are offered by the network of partners are collated as part of a common repository that consists of information such as their eligbiilty criteria or target audience and location. Programmes are categorised based on the needs and interests (such as physical, financial, social, emotional, intellectual and vocational), and intensity for those involving physical activity to facilitate referrals to suitable programmes.
4. **Collective care plans**. Especially for residents with health and social issues, case discussions led by the anchor partner, primary care and CHT are conducted to co-develop a care plan. The care plan ensures that partners are aware of the follow-ups required to address the needs of each resident, and role and accountability of each partner. This is done using terminology based on the Omaha System and documented using a standardised Combined Case Discussion template to be shared across the partners.
5. **Processes and enablers facilitating last mile care delivery**. Besides addressing immediate needs identified, case discussions provide a platform for the network of partners to collectively continue with monitoring the progress and compliance to care plans. This allows arising needs to be identified early and for care plans to be tweaked accordingly to ensure care continues to be holistic across the residents’ life journey to the end.

Our first test-pilot of the CoC model started with the Kebun Baru Integrated Dementia (Home-based) Assisted Living project (IDeAL@115) at a block of public rental housing for low-income citizens, and anchored by Dementia Singapore and the local grassroots citizen committee for the Kebun Baru neighbourhood [24]. CHT, Ang Mo Kio Polyclinic, and St Luke’s Eldercare Kebun Baru Centre were also involved as part of the partners’ network. Outreach was conducted by Dementia Singapore and the local grassroots committee, and a group of frail and vulnerable residents with multiple comorbidities were picked up and reviewed by CHT for further needs assessment. The initial reviews identified 25 residents with complex cognitive and physical health problems, and regular case discussions were carried out to co-develop and review their care plans alongside Ang Mo Kio Polyclinic. The collective care plans developed ensured an alignment across these partners on the follow-ups required to address the needs of these residents, and as well as each stakeholder’s role. Tele-consultation was started to better facilitate co-management of health needs. Medical reviews were conducted by Ang Mo Kio Polyclinic, and CHT and Dementia Singapore and the local grassroots citizen committee monitored the residents on their compliance to medical advice. As part of the collective care plans, CHT also facilitated the referrals of six residents with functional issues for day rehabilitation services nearby through an established direct access workflow with St Luke’s Eldercare Kebun Baru Centre. Residents with financial difficulties were further linked up with social service agencies through the efforts of Dementia Singapopre and the local grassroots citizen committee. Appropriate interventions were also co-designed to promote healthy living. Exercise programmes in common areas and dietary modifications in the menu provided by the community kitchen as part of the communal dining programme formed part of the One Menu of Programmes for residents in the community. To ensure last mile care delivery, these multi-sectoral collaborations have continued, with Dementia Singapore and the local grassroots citizen committee playing the role of anchor partner that continues with long-term monitoring of the collective care plans and flagging of any new, arising needs. The network members meet up regularly to discuss workplans according to the changing needs of the residents, ensuring they continue to age well in place.

### Function and Form of a CoC

The IDeAL@115 pilot allowed us to work through the initial teething issues and surfaced the possibilities to shift providers’ mindset from providing transactional, episodic care towards joining up care as a network. These provided important lessons as we continued to build CoCs across Central Zone. We summarised these in Table 1, using the Context and Capabilities for Integrating Care (CICC) Framework. Developed and validated through a literature review and qualitative interviews with leaders and care providers involved in integrated care, the CICC provides a conceptual framework of organisational factors influencing integrated care efforts [25]. This framework will guide our description of the key drivers in developing CoCs, as well as their function and form today.

**Table 1:** Matrix of function and form of CoCs.


MOTIVATING NEED	CAPABILITY (CCIC)	CORE FUNCTION	FORM (*ITEMS UNDER DEVELOPMENT*)

**1. Organisational integration of care**			

Health and social care providers work in silo, making care fragmented	Organisational/network design	Establish a network of partners offering varied services, facilitated by identifying a committed anchor NGO partner to coordinate the network to deliver needs-stratified services for residents in a defined neighbourhood	TTSH plays a developmental role in building the network, establishing the terms of engagement to organise partners for coordinated health-social care delivery, which may be handed over to an anchor organisation in the stable state Partners provide services relevant to a range of biopsychosocial needs, such as resident outreach activities^4^, preventive care services, healthy lifestyle programmes, opportunities for socialisation, befriender services, chronic disease care, transitional care The anchor NGO partner serves as the centralised point of contact for the other partners in the neighbourhood

	Accountability	Formalise relationships within the network of partners to ensure delivery of care aligned with objectives	Partners sign agreements to formalise agreed objectives, organisational responsibility and services to be delivered, and outcomes of collaborations

**2. Coordinated population health approach**			

Lack of consolidated and up-to date information on services available to service providers within a neighbourhood	Resources	Establish a repository of various services across the health-social continuum available within a neighbourhood for easy reference by providers	Each CoC collates a repository (menu) of services and programmes (addressing physical, financial, social, emotional, intellectual, vocational needs) within the neighbourhood, stratified by “generic needs” and “specific health needs” Partners access a common platform consolidating up-to-date information on programmes across all providers, including location and eligibility criteria, to facilitate referrals of residents to relevant programmes

At-risk individuals remain unknown to the health-social system	Information technology	Establish neighbourhood-based registries with standardised information for access by different partners and to track health of each HDB block	One Resident Health Record is leveraged on to keep track of social and health data of residents

	Delivering care	Identify “unknown” and “vulnerable” populations previously unknown to partners for needs assessment	Joint, targeted outreach to “unknown” and “vulnerable” residents is conducted through community health screening and door-to-door outreach

**3. Comprehensive health-social care**			

Health and social care providers conduct independent needs assessments; no provider has a complete picture of the biopsychosocial health status of residents living in neighbourhood	Delivering care	Perform biopsychosocial needs assessment as a holistic assessment of residents’ current and potential problems	Common needs assessment tools based on interRAI, such as Community Screener Tool (CST) used by AACs and the Preventive Health Visit (PHV) surveys used by SGO, have been aligned with similar screening items and post-screening follow-up workflows Care plans are co-developed using the terminology based on the Omaha System through regular case discussion platforms and shared across partners using a standardised Combined Case Discussion template

Care is episodic and often not planned or carried out over time across service providers	Delivering care	Identify services in the health-social care continuum to meet residents’ assessed biopsychosocial needs and ensure affordable access to recommended services or programmes across population segments	Partners develop collective care plans based on assessed multi-dimensional needs and preferences with a long-term view Partners take ownership of care delivery for each case and ensure delivery of intended care plan in a coordinated manner, with agility to tweak care plans as needs change

Lack of communication and coordination across health and social care providers	Delivering care	Co-develop structured protocols to plan and coordinate needed care across partners, time and settings, especially for residents with complex needs	Standardised workflows for referrals and documentation are established amongst partners, overseen by the anchor organisation Partners hold regular case discussions and facilitate referrals based on needs

	Information technology	Create an infrastructure to exchange information for timely and secure information flow	Partners are notified of residents’ hospital admissions and discharges via Central Health Linkup (facilitated by TTSH’s Healthcare Intelligence (HI) system) Partners communicate and update on care plans and interventions via email, TigerConnect (a secure and direct messaging platform) and case discussions

**4. Resident-centred care**			

Resident’s values and preferences are less considered in their care plans; lack of provider-resident relationship based on mutual responsibility and trust	Physical features	Establish a centralised touchpoint within the neighbourhood for residents to seek information or address any arising needs	The anchor organisation provides a centralised and familiar access point for residents

	Focus on patient- centredness & engagement	Take into account residents’ values, needs, and preferences in design of care plans Foster relationship-based care with an orientation to whole person care Establish peer support networks to facilitate social connection to support sustaining good health behaviours.	The anchor organisation advocates on behalf of the resident and family in health activation, case discussions and care planning CHT introduced ‘What Matters to You (WMTY)’ conversations^5^ to enable a shift towards relationship-based care; this was also extensively adopted by SGOs as an engagement framework Health coaches guide residents in addressing identified health goals Health coaches work with anchor organisations to provide group-based programmes to promote peer learning and social support amongst residents Skills-based programmes are conducted to engage residents and increase health literacy and translation of knowledge to self-care

	Resources	Embed link workers in the neighbourhood to facilitate residents’ access to programmes and services	Health coaches^6^ and primary care coordinators (PCC)^7^ link residents with relevant services and resources based on their health plans and goals Health coaches also nudge and monitor residents for actualisation of the recommended health plans


## Scaling Up of CoCs

As the anchor hospital for Central Health ICN, Tan Tock Seng Hopsital (TTSH) expanded its mission beyond the hospital walls and provision of acute care to take on a bigger role in networking building and driving the actualisation of this place-based care model. The Division for Central Health (DCH) was established in 2017 to serve this purpose.

**Figure 3:**
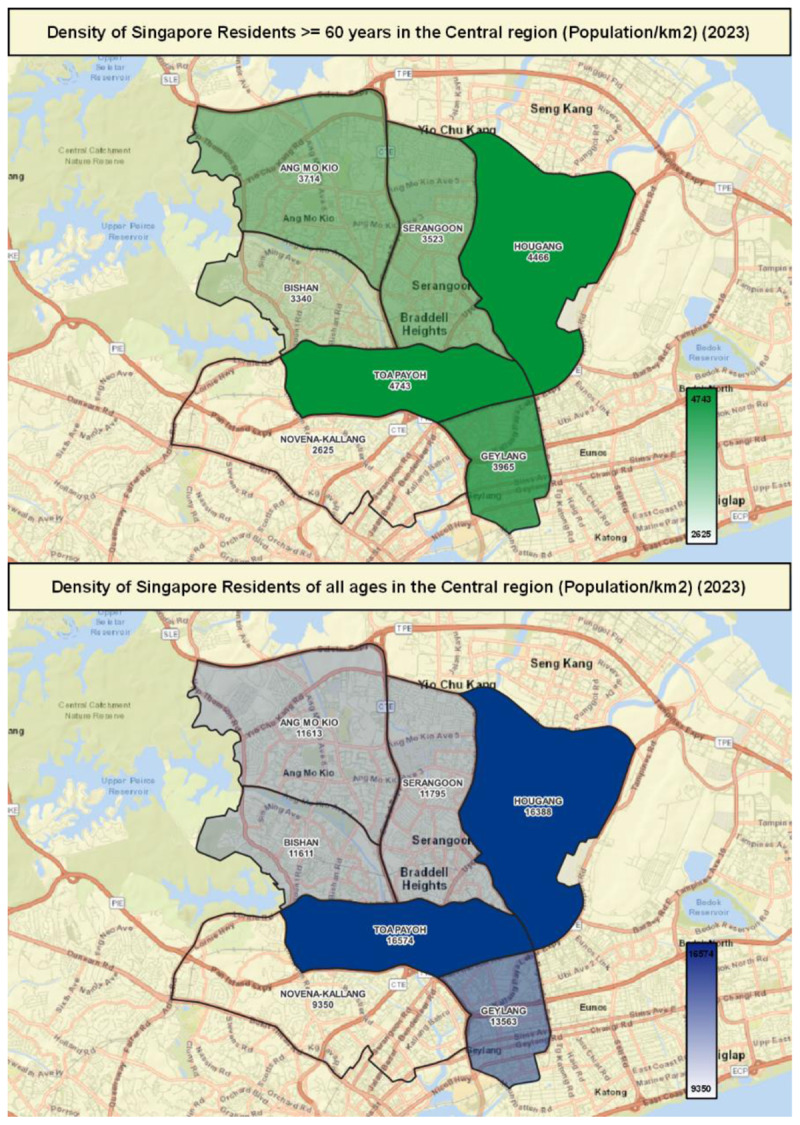
Dermarcation of Subzones by the Singapore Urban Redevlopment Authority (URA) and Population Density based on data from the Singapore Department of Statistics [26].

TTSH’s seven subzonal CHTs, each servicing a geographical boundary (or subzone) demarcated by the Singapore Urban Redevelopment Authority (URA) (Figure 3), were also re-organised under the purview of a multidisciplinary layer of team leads to drive these efforts at the ground, neighbourhood-level to build CoCs.

The selection of sites of CoCs within each subzone were further influenced by the location of communal spaces serviced by identified, willing anchor partners (usually an AAC) and based on the planning parameter of each CoC servicing a boundary with 1,000 to 4,000 seniors.

Since the first CoC was piloted with IDeAL@115 in 2020 and up to March 2023, a total of 76 provider organisations have been engaged as part of 47 CoCs. This is 70% of the projected 67 CoCs that will be required to cover the entire population of Central Zone. Since 2021, the CoC networks have reached out to approximately 21,300 residents through joint outreach and of which, 90% have been referred on for interventions or monitoring by suitable partners based on the needs identified. Separately, 416 residents have been surfaced for joint case discussions across 20 established regular platforms. An integrated list of more than 90 community-based health and social activities and programmes has also been collated to facilitate access to community resources and support residents’ health goals and plans.

The development and scaling of CoCs are not without challenges, as we next summarise some of our key learnings from across CoCs that have been developed to varying degrees of maturity. The facilitating factors, barriers and alternatives were summarised through feedback gathered from interations with partners of the Central Health ICN’s at various platforms, ranging from leadership-level meetings to working-level discussions. These were documented in the form of notes of meetings for the purposes of process improvements and improving organisation of care.

## Facilitating Factors

### Standardised workflows to link health and social care partners

The development of standardised workflows for referral, case escalation and case discussions were imperative to establishing terms of engagements amongst partners of the CoC networks. It provides documented guidelines for how partners may interface to address a range of residents’ needs, especially where one may be limited in resource or provide expertise in particular domains. This forms the basis for joining up care through the collaborative efforts of the CoC network. While we hope to ensure residents’ health and social needs are met holistically, we occasionally identified issues that were beyond the means of the network to resolve. This is especially so for vulnerable seniors with limited mental capacity or who have refused consent to seek help. In such instances, it is important for the CoC network to agree on the prioritisation of the issues that the network would like to address, and for periodic reviews of its workplans for the neighbourhood.

### Time needed to negotiate roles

Many partners were skeptical in the initial phases, and especially so when there was no additional funding or resources provided to develop CoCs. Despite these constraints, we have observed a shift in the last two years as partners have started to establish better understanding of each others’ scope, their roles and the possible contributions vis-à-vis another provider. Larger organisations have reviewed their strategic leadership plans and have become more committed to facilitate the establishment of partnerships between the healthcare and social care sectors in the communities. Smaller anchor organisations with lesser resources have contributed by organising smaller-scale projects in the neighbourhoods.

### Engagements at various levels

Close engagements at various levels was noted to be crucial in driving the development of CoCs [21]. The Central Health Leadership Council (CHLC) comprising Chief Executive Officers or the equivalent from various organisations convenes regular meetings, bringing together these key stakeholders of the Central Health ICN to set and align the strategic priorities with the ultimate aim of working together to improve the health of residents, culminating in the establishment of CoCs at the ground level. At the middle management level, points-of-contact from the various organisations work closely to translate strategies and priorities set at CHLC to execution and provide oversight of the development of CoCs across the various local communities. This also allows for agility to trial and tweak small-scale pilots based on ground-up needs sensing. The CHT team leaders further contribute to the collaborations within the networks by holding a wider lens beyond the interests of any single organisation and facilitating alignment amongst partners through mutual understanding and agreement. The progress and challenges of pilots and CoCs are updated at CHLC for further inputs and decisions at the ICN-level. Lastly, annual engagement platforms such as the Central Health Activation & Learning Kampung (CHALK) allows for the stakeholders of the Central Health ICN from ground to senior management levels to congregate in conversations. Besides networking, this provides an important platform for sharing and recognising best practices from individual CoCs, and celebrating the successes of the ICN to encourage continuous improvement, spread and scale across Central Zone.

## Barriers and Alternatives

### Segregation of health and social data

Within each CoC, we have faced with the challenge of healthcare and social care data being housed and protected within each organisation’s data protection policies. The ability to overlay data is crucial to identify residents who may be vulnerable or have little awareness of health deterioration, to design suitable programmes and provide appropriate interventions to prevent further decline [27]. For example, the massive outreach efforts by AACs and SGOs have collated much information such as the social status, living and caregiving arrangements of seniors that would be useful to profile the neighbourhood but the data sharing processes are cumbersome and hence limited. Although some attempts have been started to profile each CoC, we have not yet reached an automated process that will allow a comprehensive and most up-to-date view of residents’ needs, including social determinants of health indicators. We are in the process on working out the possibilities and ways to overcome these challenges at a more systems-level.

### Work-around misaligned policies

As CoCs mature, the network of partners will work towards developing core workflows based on other arising needs. We have witnessed the increasing complexity of partnerships as the focus of the CoC starts to shift beyond physical health and elderly care, and with mental health and family social care coming into play. Interfacing with newer partners from other unfamiliar sectors, including non-healthcare government agencies in place-based CoCs poses another challenge. For example, there has been challenges in managing residents with mental health needs due to a misalignment in policies with regard to sharing of mental health information vis-à-vis the need for access to information for a more holistic care plan to be developed.

### Geography as a constraint

Our current place-based CoCs were defined by geographical boundaries and residential addresses of residents. This presents another challenge as the movement of people in Singapore was very fluid, owing to the small size of the country. Thiam et al similarly pointed out that people live in an environment with its perceived or conceived features and dynamics such that the concept of local area has several dimensions e.g. geographical local area, lived local area, perceived local area and designed local area [28]. While we recognise the dynamic movement and social participation in their communities, some of our contextual and system problems such as our ambulance conveyance patterns to nearest hospital, patients’ choice or health seeking behaviour were difficulties that we have to overcome.

## Discussion

We have seen how health and social organisations of various capabilities come together in their own ways to meet the health and social needs of the residents in the CoCs. Such stories were similar to those of Torbay’s on how local leaders persevere in overcoming obstacles and challenges, while remaining faithful to the common goal of improving outcomes [21]. The development of workflows and processes to facilitate health and social integration has been instrumental in building the foundation in new CoCs . These worfklows were drawn in broad strokes such that teams can easily localise and contextualise them in the neighbourhoods [29]. This has challenged the linear systems that healthcare systems were traditionally built on, and efforts were required to promote these emergent ways of working as partners adapt and change constantly when they meet with different situations [30]. Some guiding principles in managing the various complex CoCs will be helpful to achieve the intended outcomes [30,31], and the development of leaders and staff to work within complex integrated care systems for population health is of upmost importance.

We also see that more mature CoCs are beginning to take the form of complex adaptive systems (CASs) characterised by more dynamic and multi-stakeholder interactions, and where traditional managerial models may be counter-productive [30]. While our earlier works in the CoCs were with AACs and anchor organisations targeted at seniors aged 60 years and above, some of the CoCs are moving towards multi-stakeholder collaborations with more partners and GPs for other age groups and population segments such as mental health and family social care. One of our upcoming works will focus on how caregivers can be supported in our CoCs. A local study on 278 family caregivers in Singapore has surfaced an elderly caregiving profile (mean age of 61.7 years), with a quarter of them (26%) not receiving any help from any family member or foreign domestic work, and only 5% of these caregivers have received any relevant trainings for caregiving [32]. Besides the low access to caregiving programmes or respite services, we also observed that some of these existing programmes and respite care may target caregivers for persons with specific conditions. Other problems after the completion of such programmes are the paucity or sustainability of supportive networks, especially in the protracted, long journey of caregiving. We are, however, at the early stages of engaging and supporting residents, caregivers and/or volunteers as part of the CoC development, to engage them as peer support leaders, and co-designing programmes that will address community health needs or caregiving support.

A scoping review also identified interventions that focused on intersectoral action and partnerships important to support population health elements and work on the social determinants of health [33]. While this is yet to be researched on in the CoCs, this multi-sectoral team working together to solve local health and social problems has been well-received by staff, with less frustrations of residents falling through the gaps. The next step is to bring this inter-sectoral work beyond the case level to one that resources and control can be delegated and shared across partners at the planning and delivery level [34]. While there are increased funding to support the AACs to support the care of seniors in their respective assigned geographical areas, further changes to the healthcare financing model to support team-based care and integrated care work will need to happen [31]. Torbay Care Trust has achieved this both at the commisssioning and whole-system governance level [21] while South East London Integrated Care system has an approach in enabling collaboration through shared incentives and aligned transformation funding [35]. These are worthy of learning in our journey to develop appropriate programmes and initiatives to improve health and social care for residents in the CoCs.

Some health or social NGOs may determine their area of service coverage by boundaries and these may differ across organisations. There could be multiple providers of similar services within the same neighbourhood and in other instances, some services might not be accessible to certain pockets of the population. Such challenges were commonly reported in integrated care systems when there were tensions between being responsive to local needs and the need for an appropriate degree of organisational consistency [34]. However for the past two years, we have observed shifts in some partners towards a more place-based care strategy, undergriding the tensions between organisational priorities and the needs of a CoC. Thus, the true success of each CoC will be dependent on the senior leadership of the partners as well as local ground staff and volunteers. We have attempted to work around existing limitations and challenges by seeking mutual agreement and aiming towards health-social care planning for residents. Further research is warranted to study the contextual or system factors and the level of partnership that may hinder or promote integration of care in the CoCs.

We are also exploring how the performance of a CoC can be better evaluated so that we will not veer towards meeting organisational or national targets at the expense of meeting local needs [34]. The evaluation should also take into consideration the uniqueness of each neigbourhood and the available partnerships and inter-sectoral support. We are now looking at the sharing of health and social care data more effectively amongst stakeholders in the CoCs so that early planning of programmes and services or tracking of health trends can be easily carried out. Besides sensing residents’ needs in the neighbourhoods, making appropriate referrals to a menu of programmes by various providers in the CoC is another piece of work in which we have made some progress. We intially collated this manually, and are now moving to the next stage of using an electronic platform (i.e. the Health Kampung mobile application) so that residents, partners and General Practitioners will be able to access programmes closer and more easily in the neigbhourhoods [36].

## Conclusion

The CoC framework has been important in driving the shift of community care to a place-based model in the Central Zone of Singapore. Many iterations have since taken place but the basic framework has remained largely intact. Changes and improvements have taken place to evolve this model, with the efforts of many committed partners in the CoCs. Not forgetting the earlier motivating needs at the beginning, we will continue to move towards a value-based localised model while balancing the pace of development of the health system vis-a-vis local needs. Different maturity and complexity levels of CoCs have been identified. Next steps for these complex CoCs will be to improve the milestone measurements to establish a possible evaluation and performance framework, and facilitating the interactions amongst the partners within each CoC to allow more resident-centric and place-based activities to take place.

## Footnotes

A multi-disciplinary team of nurses, doctors, allied health professionals, and health coaches set up by Tan Tock Seng Hospital (TTSH), the anchor hospital for Central Zone, to provide community-based services as part of its population health mission.

A drop-in centre funded by MOH through the Agency for Integrated Care (AIC) to service 3,000 – 4,000 seniors within an assigned boundary. Services include: Active Ageing Programmes; Befriending and Buddying; Information and Referral to Care Services; Vital Signs Monitoring; and connecting to Social and Lifestyle Interventions. They were previously known as “Senior Activity Centre (SAC)”.

An outreach arm of AIC that engages seniors (60 years old and above) to understand their aspirations and healthcare needs and connect them with the relevant care and support services.

Common resident outreach activities includes neighbourhood celebratory events during festivities and health screening events.

‘What Matters To You’ conversations is a framework developed by TTSH’s psychologist. It guides providers to engage with an intent to find out what residents care about and their priorities, and enables providers to work collaboratively with residents to tailor interventions and motivate them towards positive behavioural changes.

Health coaches anchor the provision of lifestyle intervention activities within the TTSH CHT. They enable residents for health and wellness maintenance by co-building health awareness and co-creating care plans with residents, coaching for lifestyle changes and compliance to care plans, and coordinating/ signposting residents to the relevant community resources to achieve their health goals.

PCCs are coordinators embedded within primary care to support primary care providers in social prescribing.

## Lessons learned

- Evolvement of CoCs towards the 3 ‘A’s and focusing on the unique health and social needs of the residents in the neighbourhoods
- The growth of the ‘neighbourhood’ team that have partnerships and collaborations beyond the current linkages and working on common health and social needs
- The participation of residents in the CoCs and developing a sense of ownership to improve their health
- Evaluation of the various CoCs with the use of appropriate measurement tools and data while recognising the uniqueness of each neighbourhood.
